# Utilizing Mixed Methodology to Increase Cultural Competency in Research with Transgender and Gender Nonconforming Individuals

**DOI:** 10.1089/trgh.2019.0057

**Published:** 2020-03-16

**Authors:** Kaitlin J. Portz, Anna Burns

**Affiliations:** ^1^ Siteman Psychology Service, Siteman Cancer Center, Barnes-Jewish Hospital, St. Louis, Missouri.; ^2^ Department of Social Work, Psychology, and Counseling, Alabama A&M University, Normal, Alabama.

**Keywords:** cultural competency, gender nonconforming, mixed methods research, pilot research, TGNC, transgender

## Abstract

Transgender and gender nonconforming (TGNC) individuals experience increased risk to mental and physical health concerns based on minority stress variables, including discrimination, internalized stigma, and expectations of violence. Research in this area displays a disconnect between provider and TGNC individuals seeking care. This study sought to improve cultural competency in research with TGNC individuals, with the ultimate goal to further explore cultural competency factors in work with TGNC individuals in research and clinically. Mixed methods research was conducted with trans masculine, trans feminine, and nonbinary identifying individuals to pilot survey measures before administration in a larger study.

## Introduction

Based on lived experiences and internal stressors, transgender and gender nonconforming (TGNC) individuals may face increased risk to a number of mental and physical health concerns. Moreover, research suggests that health care providers may not adequately address the unique needs of this population. In clinical interactions with minority groups, special care must be provided to address issues central to their lives, utilizing compassion, respect, and empathy. To this end, providers must employ cultural humility with patients, going beyond familiarity with standards of care, and working from a place of deeper curiosity and individualized patient-centered care.

These concepts must also be utilized in research to ensure sound methodology to address issues that are central and culturally relevant to TGNC concerns. This may be accomplished through piloting research utilizing mixed methodologies, including qualitative and quantitative methods, to assess cultural considerations that are relevant when working with a particular population. In addition, this may be especially vital to inform researchers who do not share common lived experiences of their research population of interest (e.g., a cisgender researcher surveying TGNC participants).

Thus, this study outlines a piloted mixed methods research design that was conducted as a precursor to a larger study, with the intent to assess cultural considerations of measures and procedures that would be utilized within a larger study. This short report summarizes (1) quantitative survey selection, (2) qualitative interviews and/or focus groups with TGNC participants, (3) qualitative thematic analysis and results, and (4) limitations and future directions.

## Literature Review

Understanding the cultural norms of any target group is vital to the research process, thus utilizing strategies to maximize effective communication is key. Wardale et al. created a modified toolkit for qualitative interviews with culturally diverse populations, including utilizing focus groups to relate positively across cultural groups.^1,2^ This toolkit formed the foundation of the interactions within this study, including use of strategies such as collaborating with a cultural insider, gaining education on cultural concerns and topics, practicing cultural humility, respecting confidentiality, displaying long-term commitment, and acknowledging the importance of fluidity and flexibility. These strategies, among others discussed by Wardale et al., are essential when building rapport and connecting with groups facing social stigma, such as TGNC individuals, who may be more reluctant to trust an outside researcher.^1,3^

In addition, in examining the cultural context of the gender-diverse population, not only do TGNC experiences differ greatly from that of sexual diverse populations, but also TGNC narratives vary widely themselves.^4^ Worthen discussed the importance of examining differences in sexual and gender-diverse identities as well as exploring intricacies in subcategories within the population.^5^ The TGNC subgroups, including trans masculine, trans feminine, and nonbinary, each face different challenges and experiences, creating a need for further examination and analysis as unique groups.

Adams et al. reviewed ethical considerations in trans health research and developed guidelines in work with this population.^6^ The researchers' guidelines inform the importance of mixed methods research with TGNC individuals, including suggestion of partnership with community stakeholders at all stages of the research process. In addition, the researchers recognize the importance of language in research with the TGNC community and suggest “nonstigmatizing” language in research. Mixed methods piloted research with TGNC individuals allows for evaluation of the cultural sensitivity of a project, involving community stakeholders who may, in ideal conditions, openly and without influence comment on the study, and in which researchers may utilize the feedback to make necessary changes moving forward in ethical study design and implementation.

## Brief Overview

This research was designed to act as the pilot phase of a larger research effort centered around utilizing the Minority Stress Model to better serve a TGNC population.^7,8^ To this end, this study sought to conduct three focus groups organized by self-identified gender identity category, informed by the literature highlighting the unique narratives and experiences between groups. The primary researcher sought to recruit groups of TGNC individuals who self-identified as trans masculine, trans feminine, and nonbinary genders for participation in focus groups. Participants were recruited within the “Deep South” United States, where access to services are limited and challenged for TGNC individuals. Participant recruitment occurred by posted flyers in two local LGBT-focused health care clinics. Participants self-referred for study involvement and completed a 30-min survey privately before arrival to meeting with the researcher. Participants were then interviewed in their respected groups, where *n*>2, in a semistructured manner about their experiences during testing. However, due to limitations in recruiting TGNC participants in the Deep South, there were several barriers to group recruitment. See Perez-Brumer et al. for additional reading in this area.^9^

The purpose of this preliminary pilot research was to assess both the acceptability and utility of the chosen assessment measures in a culturally competent manner when engaging with a TGNC population. The results from the focus groups guided the research to make any necessary changes to the overall study to ensure that the testing was completed both respectfully and accurately with a TGNC population before starting recruitment for the larger study, in which minority stress factors would then be explored.

## Methods and Analysis

This mixed methods pilot study included quantitative surveys administered through Qualtrics as well as in-person qualitative interview or focus group meetings with the primary researcher and trained research assistant. See Appendix A1 for list of survey measures included in the quantitative survey. Measures were selected based on literature informing the larger study research questions on minority stress factors and mental health outcomes. Specifically, the primary measure of interest in the larger study, the Gender Minority Stress and Resiliency Measure (GMSR), was chosen based on its novel development in examining minority stress factors adapted to trans experiences.^10^ Additional identity-specific measures were included to assess gender identity congruence and distress.^22,23^ Finally, the researcher sought to include psychometrically sound measures of mental health outcomes, including widely used measures assessing depression, anxiety, trauma experiences and symptomatology, suicidality, substance use, and sexual risk and history.^10–21,24,25^ These measures were chosen based on literature suggesting increased risk for adverse mental health outcomes related to minority stressors, for further exploration in the larger study.

In addition, demographic variables, such as sex assigned at birth, gender identity, gender expression, sexual orientation, region, employment status, income, educational level, race, age, relationship status, and religiosity were collected. Assigned sex, gender identity, and sexual orientation were assessed using the recommended protocol of The Fenway Institute,^26^ and religiosity was examined by the Duke University Religion Index.^27^

This study recruited seven TGNC-identifying individuals to participate in focus groups separated by self-identified gender identity categories (i.e., trans masculine, trans feminine, and nonbinary genders). Participants self-referred for participation in the study through two local LGBT health care clinics. Inclusion criteria included gender-diverse identity (i.e., identifying as transgender, gender nonconforming, gender queer, agender, and bigender), being age ≥18 years, having access to a computer with Internet, and comprehension and understanding of English. All procedures were approved by the university institutional review board of the primary author at the time of the study.

Participants were contacted by telephone before assignment to a focus group to review confidentiality and group rules. Participants were then sent a unique link through e-mail directing them to a Qualtrics survey to complete informed consent and survey measures before arrival to the focus group. Participants were instructed to write down comments while taking the survey to discuss during the group.

In March and April 2017, seven participants (two trans masculine, two trans feminine, and three nonbinary) completed the online survey. Participants were invited to attend in-person focus group meetings; however, one participant was unable to attend. Thus, two focus groups and one individual interview occurred (two trans masculine, one trans feminine, and three nonbinary), and participants were provided $20 Visa Gift Cards to cover travel costs to participate.

Participants met at one time-point for a 60-min meeting designed to discuss components of the study. Meeting rules and limits of confidentiality were reviewed before beginning the meeting, and participants affirmed their consent to participate in the meeting. Participants were invited to openly share their experience taking the online survey, particularly focusing on any areas that they felt were offensive, inaccurate, or irrelevant to TGNC experiences. The survey focused on the impact of proximal stressors and resiliency factors in relation to adverse mental health outcomes, as this relates to the larger study purpose. The meetings were conducted by the primary author and a trained research assistant, and both received training in qualitative interviewing and analysis. The facilitators utilized semistructured questions attending to each measure to gain a better understanding of participant's perceptions of the survey (Appendix A2). The meetings were audio recorded. Transcripts were reviewed for accuracy and identifying information removed.

Results of the qualitative meetings yielded a handful of changes in the survey design. Qualitative transcripts were reviewed by the primary researcher and research assistant using a theme-based approach. Owing to the small size of the data set, the researchers utilized manual coding, and each independently coded the three transcripts. The researchers then met to conduct qualitative comparison of the codes and determine agreement with the coding to create a codebook based on themes that emerged in the data. Several themes emerged through participants' discussion of survey measures in the meetings. Highlighting minority stress experiences related to the larger study, participants discussed a conflict in pride and identity, feelings of lack of community as TGNC-identifying individuals living in the Deep South, and experiences of discrimination. However, these experiences did not prompt changes in survey design.

Most relevant to survey modifications included the differences in sexual experiences between TGNC and cisgender persons, as measured by the Sexual History Questionnaire (SHQ).^21^ Participants discussed gender bias in the SHQ; thus, it was determined to remove this questionnaire from further iterations of the survey, as sexual behavior and experiences were appropriately captured by the Sexual Risk Survey for the purpose of the larger study.^20^ Further research may be warranted to modify the SHQ for TGNC experiences. Furthermore, participants discussed the need for additions to the survey, including supplemental instructions in the survey for emotionally sensitive measures (i.e., “trigger warnings”) such as the PCL-5 or the GMSR, as well as an optional comment box to allow for further expression of experiences if so desired.

## Brief Summarizing Conclusion

Creating an environment of cultural understanding and competence in research is a worthwhile goal in the field. To that end, it is critical that researchers further examine and find ways to combat their own biases and limitations in working with culturally different populations. Use of mixed methods research as a pilot study to guide further research is a prudent idea when working with minority populations, as this may assist in identifying factors that may limit research findings and perpetuate cultural microaggressions or misunderstanding during the research process.

In this piloted study, barriers to recruitment resulted in low sample size, thus limiting research findings. Given recruitment in the Deep South, United States, several barriers contributed to difficulties in recruitment of TGNC individuals, including medical mistrust of providers by the TGNC community and experiences of transphobia in health care settings.^9^ Recruitment occurred in two LGBT-specific health care facilities in the Deep South; however, given these barriers, participants may have been understandably reluctant to self-refer to engage in a research study in which they openly shared their thoughts and opinions to a cisgender researcher.

Although this study served as a “jumping off point” for larger data collection of a national cohort beyond the Deep South, further mixed methods research would be helpful in determining the relevance and acceptability, as well as psychometric properties of the selected measures within a diverse TGNC population. Further research is needed to expand inclusivity of measures for use with TGNC individuals, such as sexual risk and history measures, as well as obtaining normative data with TGNC populations for measures that are considered psychometrically sound for use in the general population.

In addition, it became clear that further consideration is needed in consultation with stakeholders during all aspects of the research process, including research design, implementation and execution, and dissemination.^6,28^ Embracing a culturally competent approach to the research process, not only does research benefit from consultation with community stakeholders, but also research collaborations with stakeholders at all levels of data collection, interpretation, and writing allow for further advances in the field. Thus, although piloting research may be an initial first step in increasing culturally competent research with a TGNC population, further research utilizing stakeholders is warranted for both improving research design and increasing valid normed measures for use with this population.

## Abbreviations Used

ASSIST: Alcohol, Smoking, and Substance Involvement Screening Test
CESD-R: Center for Epidemiological Studies Depression Scale-Revised
DASS-21: Depression Anxiety Stress Scales-21
GAD-7: Generalized Anxiety Disorder 7-item scale
GMSR: Gender Minority Stress and Resiliency Measure
INQ: Interpersonal Needs Questionnaire
LEC: Life Events Checklist
MEPOS: Measure of Episodic Planning of Suicide
Mini-SPIN: Mini-Social Phobia Inventory
PCL-5: PTSD Checklist for DSM-5 with Extended Criterion A
SHQ: Sexual History Questionnaire
SRS: Sexual Risk Survey
TCS: Transgender Congruence Scale
TGNC: transgender and gender nonconforming

## Appendix

**Appendix A1.** List of Survey Measures

Measure
Gender Minority Stress and Resiliency measure (GMSR)^10^
Generalized Anxiety Disorder 7-item scale (GAD-7)^11^
Mini-Social Phobia Inventory (Mini-SPIN)^12^
Depression Anxiety Stress Scales-21 (DASS-21)^13^
Center for Epidemiological Studies Depression Scale-Revised (CESD-R)^14^
Life Events Checklist (LEC)^15^
PTSD Checklist for DSM-5 with Extended Criterion A (PCL-5)^16^
Interpersonal Needs Questionnaire (INQ)^17^
Measure of Episodic Planning of Suicide (MEPOS)^18^
Alcohol, Smoking, and Substance Involvement Screening Test (ASSIST)^19^
Sexual Risk Survey (SRS)^20^
Sexual History Questionnaire (SHQ)^21^
Transgender Congruence Scale (TCS)^22^
Sexual Identity Distress Scale (modified by primary author for TGNC populations)^23^

**Appendix A2.** Qualitative Meeting Question Guide

1. Were there any questions that were unclear or did not make sense? Please explain.
2. Were there any questions that made you feel uncomfortable? Please explain.
3. Were there any questions that were offensive? Please explain.
4. Did the demographic questionnaire accurately reflect your demographics? Please explain.
5. Were there any areas that you felt were largely missing from the GMSR to accurately reflect any experiences you may have had related to discrimination, rejection, or harassment?
○ With regard to connecting to your community or pride in your identity?
○ With regard to negative expectations of the future, internalized beliefs, or nondisclosure?
6. Were there any concerns or difficulties with:
○ GAD-7?
○ Mini-SPIN?
○ DASS-21?
○ CESD-R?
○ PCL-5 or LEC-5?
○ INQ?
○ MEPOS?
○ ASSIST?
○ SRS?
○ SHQ?
○ DUREL?
○ TCS?
○ GID?
7. Any general comments or suggestions for edits to the survey?
8. How long did it take to complete from beginning to end?
